# Supplementary figures and images for: Examining the controllability of sepsis using genetic algorithms on an agent-based model of systemic inflammation
Source: PLoS Comput Biol. 2018 Feb 15;14(2):e1005876. doi: 10.1371/journal.pcbi.1005876 (PMC5813897; doi:10.1371/journal.pcbi.1005876)

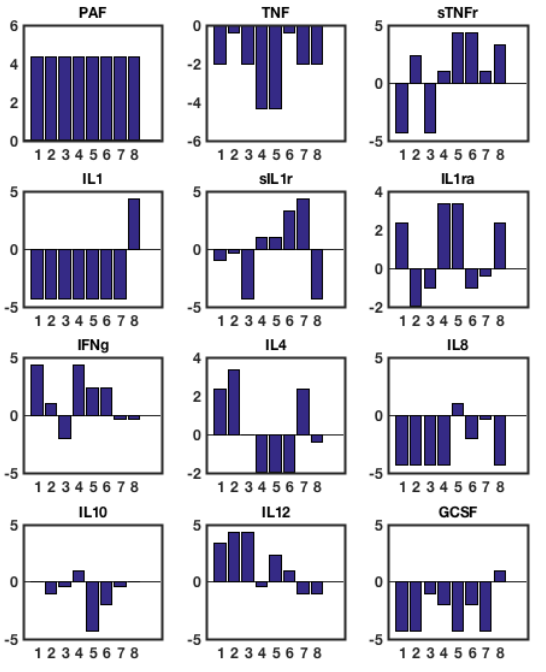

Supplement: S1 Fig — This figure contains 12 bar graphs, each of which represents the sequence of augmentations/inhibitions applied to a particular cytokine pathway. The intervention sequence is shown on the x-axes and the base-2 logarithm of the augmentation or inhibition strength is shown on the y-axes. This sequence of interventions lowered the probability of death from 68% to 12% for the patient upon which the GA was trained; the probability of death was lowered from 82% to 16% for the general population using an identical parameter set. (TIFF) [file pcbi.1005876.s001.tiff]
